# Identification of extracellular matrix-related biomarkers in colon adenocarcinoma by bioinformatics and experimental validation

**DOI:** 10.3389/fimmu.2024.1371584

**Published:** 2024-04-17

**Authors:** Yongkui Yin, Xiaojie Yang, Zhengyi Cheng, Hui Wang, Jun Lei, Dan Wang, Peiwen Wang, Biao Li, Jing Mi, Qi Yuan

**Affiliations:** ^1^ College of Life Science, Mudanjiang Medical University, Mudanjiang, China; ^2^ Department of Pathology, Xi’an No.3 Hospital, The Affiliated Hospital of Northwest University, Xi’an, China; ^3^ Department of Rheumatology and Immunology, Tangdu Hospital of The Air Force Medical University, Xi’an, China; ^4^ Department of Assets Management, Mudanjiang Medical University, Mudanjiang, China

**Keywords:** extracellular matrix, colon adenocarcinoma, tumor microenvironment, prognosis, WGCNA

## Abstract

**Backgrounds:**

Extracellular matrix (ECM) is an important component of tumor microenvironment, and its abnormal expression promotes tumor formation, progression and metastasis.

**Methods:**

Weighted gene co-expression network analysis (WGCNA) was used to identify ECM-related hub genes based on The Cancer Genome Atlas (TCGA) colon adenocarcinoma (COAD) data. COAD clinical samples were used to verify the expression of potential biomarkers in tumor tissues, and siRNA was used to explore the role of potential biomarkers in cell proliferation and epithelial−mesenchymal transition (EMT).

**Results:**

Three potential biomarkers (*LEP*, *NGF* and *PCOLCE2*) related to prognosis of COAD patients were identified and used to construct ERGPI. Immunohistochemical analysis of clinical samples showed that the three potential biomarkers were highly expressed in tumor tissues of COAD patients. Knockdown of *LEP*, *NGF* or *PCOLCE2* inhibited COAD cell proliferation and EMT. Dictamnine inhibited tumor cell growth by binding to these three potential biomarkers based on molecular docking and transplanted tumor model.

**Conclusion:**

The three biomarkers can provide new ideas for the diagnosis and targeted therapy of COAD patients.

## Introduction

Colon adenocarcinoma (COAD) is the third most commonly diagnosed cancer and the second leading cause of cancer death worldwide, which has become a global public health challenge (1). With the development of cancer detection technology, the rate of early diagnosis has improved, but the diagnosis of COAD is rapidly shifting to younger and more advanced stage (2). It takes more than 10 years to develop COAD from polyp to adenocarcinoma, and this long progression provides an opportunity for intervention to prevent its progression into advanced stage (3). In recent years, the rapid development of high-throughput sequencing technology and bioinformatics has promoted the exploration of COAD (4, 5). Therefore, understanding the pathogenesis of COAD from the perspective of tumor molecular targets based on bioinformatics analysis is of great significance for the treatment and prevention of COAD.

The extracellular matrix (ECM) is a complex structure composed of various proteins that regulates biological functions by regulating intercellular crosstalk (6–8). ECM is an important component of tumor microenvironment (TME), and its abnormal expression promotes tumor formation, progression and metastasis (9, 10). Clinicopathological analysis has confirmed that excessive deposition of ECM in tumor patients is associated with poor prognosis (11, 12). Recently, high-throughput sequencing analysis revealed that ECM-related genes are aberrantly expressed during tumor progression (13, 14). The accumulation of ECM induces hypoxia and metabolic stress, which in turn activates anti-apoptotic and drug-resistance pathways in tumors (15). In addition, the high density of ECM obstructs the infiltration of immune cells, which affects the effect of tumor immunotherapy (16–18). Therefore, the prognostic model based on ECM-related genes will provide a basis for predicting the recurrence of COAD patients.

Leptin is the glycoprotein product of leptin gene (*LEP*). Epidemiological studies support the LEP is associated with an increased risk of COAD (19). Studies have shown that the expression level of LEP mRNA in COAD tissues is upregulated, which is associated with poor prognosis of COAD patients (20, 21). Peripheral nerves form a complex tumor microenvironment composed of multiple cell types and factors, including nerve growth factor (NGF). NGF plays an important role in the growth, invasion and metastasis of several solid tumors. Lei et al. found that NGF secreted by pancreatic cancer cells induces autophagy of schwann cells, which in turn is involved in the proliferation and metastasis of pancreatic tumors (22). Hayakawa et al. demonstrated that overexpression of NGF significantly accelerated the growth and invasion of gastric tumors (23). Procollagen C-endopeptidase enhancer 2 (PCOLCE2) is an ECM glycoprotein that acts as a functional procollagen C-proteinase enhancer (24). PCOLCE2 is involved in EMT and plays a key role in promoting COAD metastasis (25). He et al. demonstrated that PCOLCE2 is a characteristic gene affecting clinical prognosis in COAD patients based on bioinformatics analysis (26).

**Figure 1:**
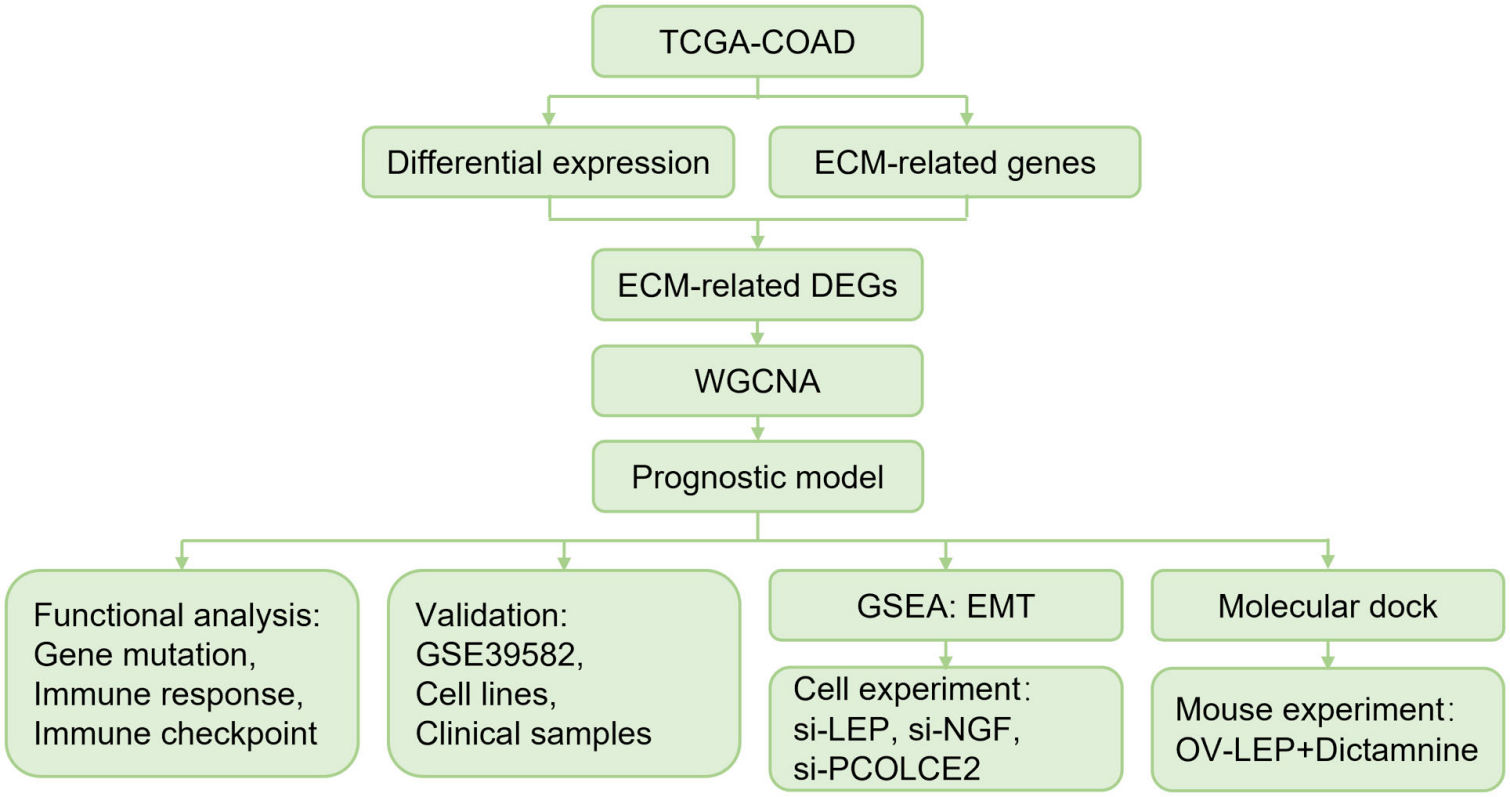
Overall schematic diagram.

In this study, we identified three ECM-related genes (*LEP*, *NGF* and *PCOLCE2*) associated with COAD prognosis by WGCNA and Lasso-Cox regression. We verified that LEP, NGF and PCOLCE2 were highly expressed in tumor tissues using COAD clinical samples. The three ECM-related genes were used to construct the ECM-related gene prognostic index (ERGPI). We found that ERGPI-low patients exhibited a positive anti-tumor immune response and better prognostic survival compared to ERGPI-high patients. Knockdown of the three genes inhibited cell proliferation and epithelial-mesenchymal transition (EMT). In addition, dictamnine could bind to these three proteins based on molecular docking, and its effect was validated in a xenograft model (**Figure 1**). The three potential biomarkers could provide new targets for the diagnosis and treatment of COAD patients.

## Materials and methods

### Data acquisition

RNA-seq data and clinical information of COAD patients were downloaded from The Cancer Genome Atlas (TCGA) database (https://portal.gdc.cancer.gov/), including 458 tumor samples and 41 normal samples. GSE39582 dataset (27) was downloaded from the Gene Expression Integrated Database (GEO) (http://www.ncbi.nlm.nih.gov/geo/) and normalized by the robust multichip average (RMA) algorithm. ECM-related genes were collected from previous literature (28) (**Supplementary Table S1**).

### Analysis of ECM-related differentially expressed genes

We normalized counts data using the “DESeq2” R package and calculated differentially expressed genes (DEGs) between normal and tumor samples (|log2(FC)| > 1 and p < 0.05). The online Venn diagram analysis tool (jvenn, http://jvenn.toulouse.inra.fr/app/index.html) was used to extract the intersection of DEGs and ECM-related genes to obtain ECM-related DEGs. The “ggplot2” and “pheatmap” R packages were used for visualization of volcano and heatmap.

### Functional enrichment analysis

Functional enrichment analysis was performed using the “clusterProfiler” R package, including Gene Ontology (GO) and the Kyoto Encyclopedia of Genes and Genomes (KEGG) analysis. The “GOplot” R package was used for visualization of GO and KEGG analysis.

### Weighted gene co-expression network analysis

The gene co-expression network was constructed using the “WGCNA” R package. The specific steps were as follows: Firstly, the optimal soft threshold power was calculated; Secondly, the adjacency matrix was constructed according to the selected soft threshold power. Thirdly, a hierarchical clustering tree was established to cluster co-expressed genes into the same module. Finally, the correlation between module genes and traits was calculated. The module with the most significant correlation was selected for follow-up research. In this study, we screened hub genes according to threshold weight > 0.1.

### Construction of ERGPI

Based on the hub genes screened by WGCNA, survival analysis was performed using the “survival” and “survminer” R packages. Significant genes affecting survival were identified by Lasso-Cox regression analysis. ERGPI was calculated by multiplying the expression value of significant genes by their weights and adding them together.

### Gene mutation analysis

COAD patients were divided into high and low subgroups based on the median ERGPI scores. Genetic alteration information was obtained from cBioQPortal database (https://www.cbioportal.org/), and the “Maftools” R package was used to analyze gene mutation.

### Gene set enrichment analysis

According to the median ERGPI score and the expression of *LEP*, *NGF*, *PCOLCE2*, COAD patients were divided into high and low subgroups. We used the “DESeq2” R package to analyze DEGs according to the high and low subgroups. The GSEA method based on epithelial−mesenchymal transition (EMT) gene set was used to enrich DEGs by using the “clusterProfiler” R package.

### Immune characteristic analysis

There are seven steps in the Cancer-Immunity Cycle, including the release of cancer cell antigens (Step 1), cancer antigen presentation (Step 2), priming and activation (Step 3), trafficking of immune cells to tumors (Step 4), infiltration of immune cells into tumors (Step 5), recognition of cancer cells by T cells (Step 6) and killing of cancer cells (Step 7) (29). We obtained a list of Cancer-Immunity Cycle genes from the Tracking Tumor Immunophenotype (TIP) website (http://biocc.hrbmu.edu.cn/TIP/), and used the ssGSEA algorithm to score the Cancer-Immunity Cycle. The correlation between ERGPI score and immune checkpoints was performed by Pearson analysis.

### Docking study

Molecular docking study was performed using AutoDock Vina. The LEP (PDB ID: 1AX8), NGF (PDB ID: 1WWW) and PCOLCE2 (AlphaFold ID: Q9UKZ9) structures were obtained from protein databank. The dictamnine structure was obtained from the PubChem database (Compound CID: 68085). The water molecule was removed from the target protein by PyMOL, and then the target protein was imported into AutoDock Tools 1.5.7 for hydrogenation, charge calculation and non-polar hydrogen binding. The binding sites of proteins were determined by Grid BOX. Finally, AutoDock Vina was run for molecular docking using CMD command characters and the results were visualized using PyMOL.

### Cell culture and transient gene transfection

The NCM460, HT29 and HCT116 cell lines were obtained from the American Type Culture Collection (ATCC; Manassas, VA, USA). The cells were cultured in Dulbecco’s modified Eagle’s medium containing 10% fetal bovine serum at 37°C with 5% CO_2_. Negative control, LEP, NGF and PCOLCE2 siRNA were synthesized by GenePharma Co., Ltd. (Suzhou, China). Negative control siRNA, (sense) 5’-CCUCGUGCCGUUCCAUCAGGUAGUU-3’ and (antisense) 5’-CUACCUGAUGGAACGGCACGAGGUU-3’; LEP siRNA #1, (sense) 5’-CCUUCCAGAAACGUGAUCCAAUU-3’ and (antisense) UUGGAUCACGUUUCUGGAAGGAU-3’; LEP siRNA#2, (sense) 5’-ACACUGGCAGUCUACCAACAGUU-3’ and (antisense) 5’-CUGUUGGUAGACUGCCAGUGUAU-3’. NGF siRNA #1, (sense) 5’-CAACAGUGUAUUCAAACAGUAUU-3’ and (antisense) UACUGUUUGAAUACACUGUUGAU-3’; NGF siRNA#2, (sense) 5’-GCGGUCAUCAUCCCAUCCCAUUU-3’ and (antisense) 5’- AUGGGAUGGGAUGAUGACCGCAU-3’. PCOLCE2 siRNA #1, (sense) 5’-CGCCAAUUGUGUCUGAGAGAAUU-3’ and (antisense) UUCUCUCAGACACAAUUGGCGAU-3’; PCOLCE2 siRNA#2, (sense) 5’-GAGUUGUGUGAAGAUGUCAAAUU-3’ and (antisense) 5’- UUUGACAUCUUCACACAACUCAU-3’. HT29 and HCT116 cells were transfected with 53.3 nM siRNA using si-mate transfection reagent (GenePharma, Suzhou, China) according to the manufacturer’s protocols.

### RNA isolation and quantitative real-time polymerase chain reaction

Total RNA was extracted by Trizol (Beyotime, Beijing, China). According to the manufacturer’s protocols, a total of 500 ng of RNA was first reverse-transcribed into cDNA using BeyoRT™ II cDNA kit (Beyotime, Beijing, China). Next, qRT-PCR was performed using the BeyoFast™ SYBR Green qPCR Mix kit (Beyotime, Beijing, China). The relative expression of mRNA was calculated using 2^(-ΔΔCt)^ method. The primers used are as follows: *LEP*, 5’-GCTGTGCCCATCCAAAAAGTCC-3’ (forward) and 5’-CCCAGGAATGAAGTCCAAACCG-3’ (reverse); *NGF*, 5’-ACCCGCAACATTACTGTGGACC-3’ (forward) and 5’-GACCTCGAAGTCCAGATCCTGA-3’ (reverse); *PCOLCE2*, 5’-GCAGTGAAGGTTTTCCTGGAGTG-3’ (forward) and 5’- AGTCATAGCGGCACAGGTTGTC-3’ (reverse). *ACTB*, 5’-CACCATTGGCAATGAGCGGTTC-3’ (forward) and 5’-AGGTCTTTGCGGATGTCCACGT-3’ (reverse).

### Western blot analysis

Western blot analysis was performed as described previously (30). Total protein was extracted by RIPA lysis buffer (Beyotime, Beijing, China) and quantified by bicinchoninic acid (BCA) method. The protein was isolated by SDS-PAGE and transferred to PVDF membrane, and incubated at 4°C overnight. For validation of potential markers, the primary antibodies for incubation of NCM460, HT29 and HCT116 cells were anti-LEP (Bioss, Beijing, China), anti-NGF (Beyotime, Beijing, China), anti-PCOLCE2 (CUSABIO, Wuhan, China). For the effect of *LEP*, *NGF* and *PCOLCE2* knockdown on EMT, the primary antibody for HT29 and HCT116 cell incubation was anti-E-cadherin (Affinity Biosciences, Cincinnati, OH, USA). The reference antibody was anti-β-actin (Beyotime, Beijing, China). HRP-conjugated goat anti-rabbit IgG was used as secondary antibody. All bands were quantified using ImageJ.

### Immunohistochemical analysis

Thirty pairs of tumor and adjacent tissue samples of COAD patients were collected from the Second Affiliated Hospital of Mudanjiang Medical University and Xi’an No.3 Hospital, the Affiliated Hospital of Northwest University. All patients received written informed consent. All protocols were authorized by the Ethics Committee of Mudanjiang Medical University (2023-MYSZR02) and conformed to the Declaration of Helsinki of the World Medical Association. The immunohistochemical analysis process was as described previously (31). Paraffin sections with a thickness of 5 μm were prepared by fixing the tissues with 4% paraformaldehyde. Overnight incubation at 4°C was performed with the following primary antibodies: anti-LEP, anti-NGF and anti-PCOLCE2. HRP-conjugated goat anti-rabbit IgG was used as secondary antibody. The sections were examined under a microscope (DM3000, Leica).

### Cell viability analysis

HT29 and HCT116 cells were cultured in 96-well plates at a density of 5 × 10^3^ cells per well and then transfected with control, LEP, NGF or PCOLCE2 siRNA after 48 h. According to the manufacturer’s protocol. The cell viability was detected by 3-(4,5-dimethylthiazol-2-yl)-2,5-diphenyl-tetrazolium bromide (MTT) method. Each well was added with 10 µL MTT (final concentration 0.5 mg/mL, Beyotime, Beijing, China) and incubated at 37°C for 4 h. Dimethyl sulfoxide (DMSO, Beyotime, Beijing, China) was added to dissolve the formaldehyde crystals. Cell viability was calculated by measuring the absorbance at 490 nm using a microplate reader.

### Mouse model of transplanted tumor

Female C57BL/6 mice (6-8 weeks old, 18-20 g) were used to construct transplanted tumor models. 1 × 10^6^ MC38 (OV-Vector or OV-LEP) cell suspension was injected subcutaneously into the abdomen of mice. 4 mice injected with OV-Vector cells were selected for intraperitoneal injection of dictamnine (50 mg/kg) every 2 days. 4 mice injected with OV-LEP cells were selected for intraperitoneal injection of dictamnine (50 mg/kg) every 2 days. 4 mice injected with OV-Vector cells were selected to be intraperitoneally injected with equal doses of PBS every 2 days. Tumor size was measured every 3 days. All mice were euthanized by CO_2_ administration. All protocols were authorized by the Ethics Committee of Mudanjiang Medical University (2021013–1).

### Statistical analysis

All data were presented as mean ± SD and analyzed using GraphPad Prism (version 9.0) and R (version 4.2.1). Student’s t test was used for variable differences between the two groups, and one-way ANOVA and multiple comparison test were used for variable differences between more than two groups. *p*<0.05 was considered statistically significant.

## Results

### Differential expression analysis of ECM-related genes in COAD patients

The differential expression of TCGA-COAD transcriptome data was analyzed. A total of 8100 DEGs were obtained in tumor samples (n = 458) compared to normal samples (n = 41) (**Supplementary Figures S1A, B**). These DEGs intersected with ECM-related genes, resulting in 493 ECM-related DEGs (**Supplementary Figures S1C, D**). Functional enrichment analysis of 493 ECM-related DEGs revealed 1049 GO terms and 34 KEGG pathways (details in **Supplementary Table S2**). The top 10 significantly enriched GO terms are shown in **Supplementary Figure S2A**. ECM-related DEGs are involved in biological processes mainly extracellular matrix organization, extracellular structure organization and external encapsulating structure organization. The main molecular functions involved are receptor ligand activity, signaling receptor activator activity, cytokine activity, extracellular matrix structural constituent and growth factor activity. The main cell component involved is collagen-containing extracellular matrix. The top 10 significantly enriched KEGG pathways are shown in **Supplementary Figure S2B**, which mainly involved Cytokine-cytokine receptor interaction, Viral protein interaction with cytokine and cytokine receptor, Protein digestion and absorption, PI3K-Akt signaling pathway, ECM-receptor interaction, Chemokine signaling pathway, Rheumatoid arthritis, IL-17 signaling pathway, Breast cancer and Gastric cancer.

### Co-expression modules of ECM-related genes

**Figure 2:**
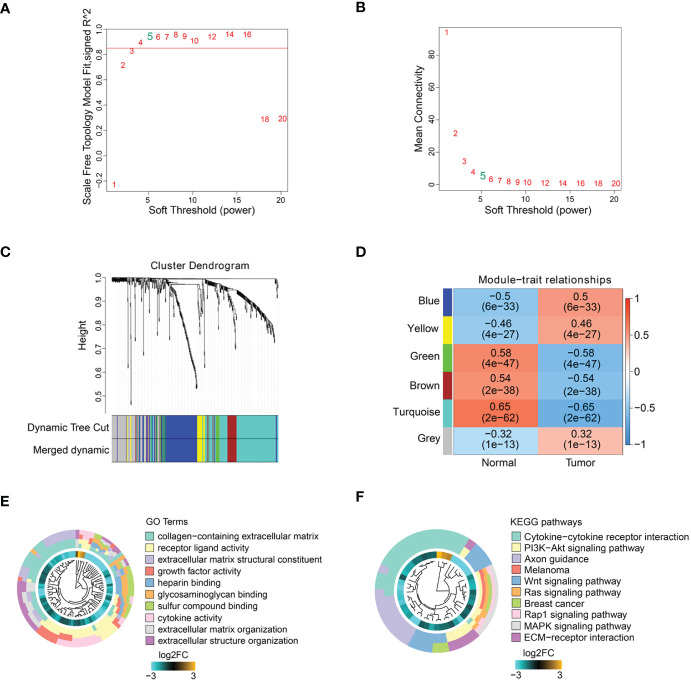
ECM-related hub genes screening based on WGCNA. **(A, B)** Scale-free fitting index analysis of soft-thresholding powers. **(C)** Cluster dendrogram. **(D)** Module-trait correlation heatmap. GO enrichment analysis **(E)** and KEGG enrichment analysis **(F)** of ECM-related hub genes.

To obtain ECM-related hub genes in COAD patients, we screened candidate ECM-related DEGs (n = 493) by WGCNA analysis. A scale-free topological network (β = 5) was established (**Figures 2A, B**). Based on the hierarchical clustering method, the co-expressed genes were divided into different modules and color-coded (**Figure 2C**). We further explored the correlation between modules and COAD patients, and plotted the module-trait heatmap based on Spearman’s correlation analysis (**Figure 2D**). The turquoise module was most closely related to COAD patients, and genes in this module were selected for further analysis (**Figure 2D**). The turquoise module contained 182 genes, of which 127 genes with a threshold weight > 0.1 probably serve an essential pathobiological role (**Supplementary Table S3**). Functional enrichment analysis was performed for these genes (n = 127), and the top 10 significantly enriched GO terms and KEGG pathways are shown in **Figures 2E, F** (details in **Supplementary Table S4**). The top 10 GO terms mainly involved collagen-containing extracellular matrix, receptor ligand activity, extracellular matrix structural constituent, growth factor activity, heparin binding, glycosaminoglycan binding, sulfur compound binding, cytokine activity, extracellular matrix organization and extracellular structure organization. The top 10 KEGG pathways mainly involved Cytokine-cytokine receptor interaction, PI3K-Akt signaling pathway, Axon guidance, Melanoma, Wnt signaling pathway, Ras signaling pathway, Breast cancer, Rap1 signaling pathway, MAPK signaling pathway and ECM-receptor interaction.

### Construction of prognostic model based on ECM-related genes

**Figure 3:**
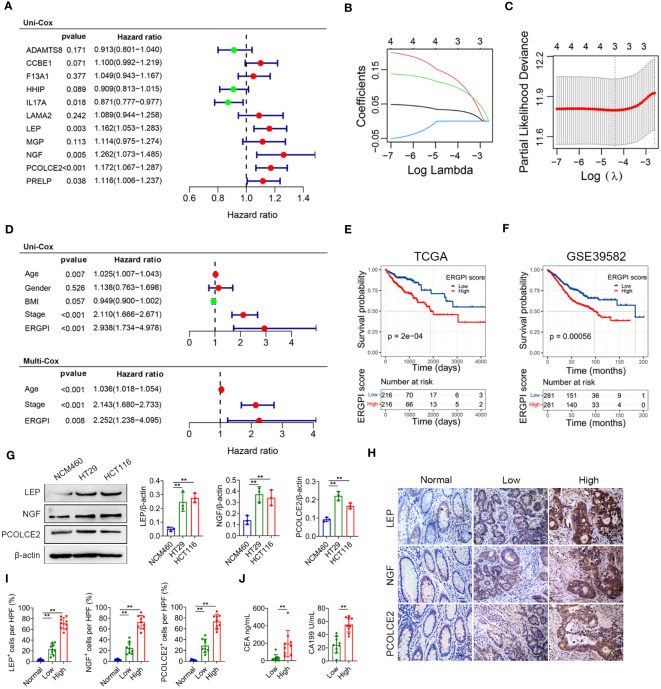
Construction and prognosis analysis of ERGPI. **(A)** Unifactorial Cox analysis of 11 ECM-related hub genes. The variation characteristics of variable coefficients **(B)** and the selection process of the optimum value of the parameter λ **(C)** in Lasso regression model by 10-fold cross-validation method. **(D)** Univariate Cox analysis of clinicopathological factors and ERGPI score, multivariate Cox analysis of significant factors in univariate Cox analysis. Survival analysis of different ERGPI subgroups in TCGA-COAD data **(E)** and GSE39582 dataset **(F)**. **(G)** The protein expression levels of LEP, NGF and PCOLCE2 in cell lines (NCM460, HT29 and HCT116) were determined using western blot analysis. The densities of protein were quantified using densitometry. LEP, NGF and PCOLCE2 were normalized to β-actin. **(H, I)** Representative immunohistochemical staining of LEP, NGF and PCOLCE2 in tumor and adjacent tissues of COAD patients (scale bar: 50 μm). **(J)** Serum CEA and CA199 levels of COAD patients. **p < 0.01.

Among the 127 turquoise module genes screened, the expression of 11 ECM-related hub genes was closely associated with poor prognosis in COAD patients by Kaplan-Meier analysis (**Supplementary Figure S3**). To construct the prognostic model, we screened five genes (*IL17A*, *LEP*, *NGF*, *PCOLCE2* and *PRELP*) from the eleven genes that were associated with prognosis by univariate Cox analysis (**Figure 3A**). We further identified three prognostic genes by Lasso regression analysis (**Figures 3B, C**). Then, the ECM-related gene prognostic index (ERGPI) of COAD patients was calculated based on the prognostic characteristics of three genes (*LEP*, *NGF* and *PCOLCE2*). The calculation formula is: ERGPI = (0.033 × expression of *LEP*) + (0.141 × expression of *NGF*) + (0.110 × expression of *PCOLCE2*). According to the median ERGPI, COAD patients were divided into ERGPI-high subgroup and ERGPI-low subgroup. Age, tumor stage, ERGPI were significantly correlated with the prognosis of COAD patients by univariate Cox regression analysis (**Figure 3D**). Multivariate Cox regression analysis further confirmed ERGPI as an independent prognostic factor (**Figure 3D**). Furthermore, COAD patients with low ERGPI scores had significantly better prognosis than those with high ERGPI scores (**Figure 3E**). The effect of ERGPI was verified using the GSE39582 dataset, and the result was consistent with the TCGA-COAD data (**Figure 3F**).

### Potential biomarkers were validated in COAD patients

To validate the three potential biomarkers, we performed experimental validation on cell lines and COAD clinical samples. The expression levels of LEP, NGF and PCOLCE2 proteins were significantly increased in COAD cells (HT29 and HCT116) compared with normal colon epithelial cells (NCM460) (**Figure 3G**). In addition, we performed immunohistochemical analysis on tumor and adjacent tissues of COAD patients. As shown in **Figures 3H, I**, the expression levels of LEP, NGF and PCOLCE2 proteins in tumor tissues were significantly increased compared with adjacent tissues. According to the results of immunohistochemical analysis, patients with high expression of all three proteins were defined as the biomarker high expression group, and patients with low expression of all three proteins were defined as the biomarker low expression group. The expression levels of carcinoembryonic antigen (CEA) and carbohydrate antigen 199 (CA199) were significantly increased in patients in the biomarker high expression group compared with the biomarker low expression group (**Figure 3J**).

### Gene mutation landscape of ERGPI high and low subgroups

**Figure 4:**
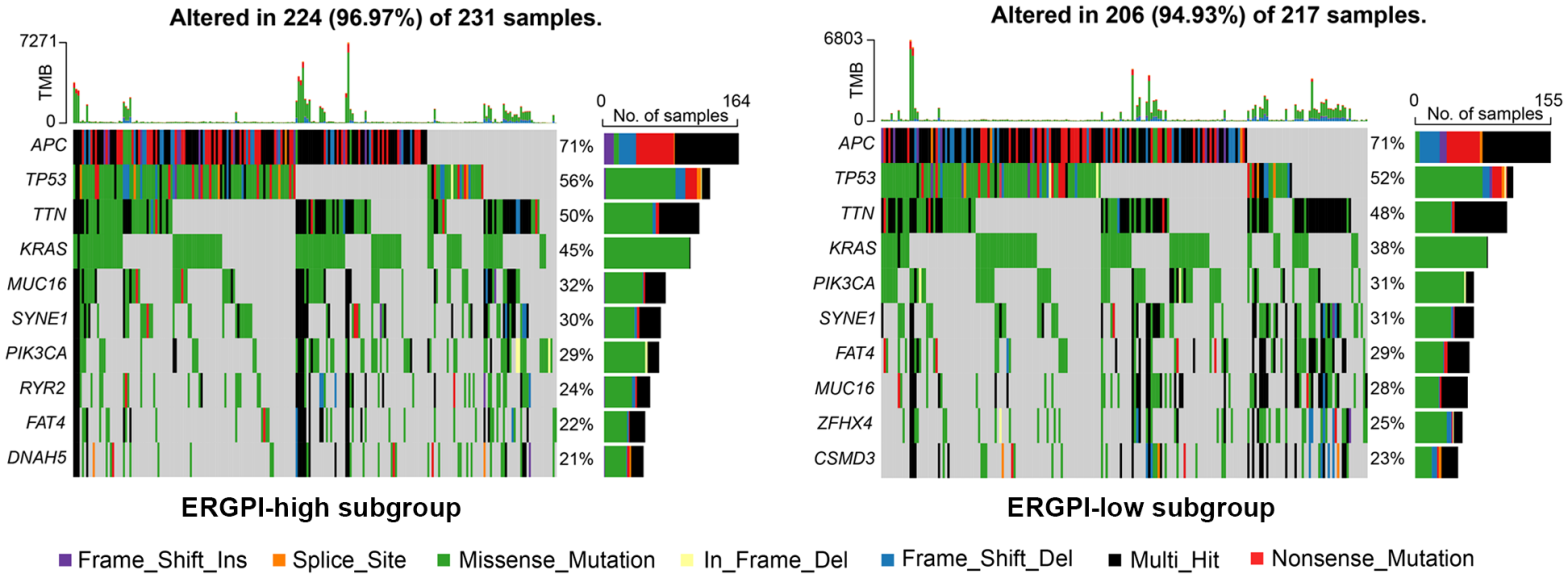
Gene mutation analysis of high and low subgroups. The top 10 significantly mutated genes of different ERGPI subgroups. Mutated genes (rows, top 10 rows) sorted by mutation rate. The arrangement of samples (columns) emphasizes the mutual exclusion of mutations. The percentage of mutations is displayed on the right, and the total number of mutations is displayed on the top. The color code indicates the mutation type.

We further explored gene mutation landscape of ERGPI high and low subgroups, the number of mutation samples in the ERGPI-high subgroup was higher than that in the ERGPI-low subgroup (96.97% vs. 94.93%). The top 10 genes with the highest mutation rate in different ERGPI subgroups are shown in **Figure 4**, where the mutation rates of *APC*, *TP53*, *TTN*, *KRAS*, *MUC16*, *SYNE1*, *PIK3CA* and *FAT4* were higher than 20% in both groups of patients. The most significant difference was *KRAS* mutation [ERGPI-high subgroup (45%) vs ERGPI-low subgroup (38%)].

### Anti-tumor immune response of ERGPI high and low subgroups

**Figure 5:**
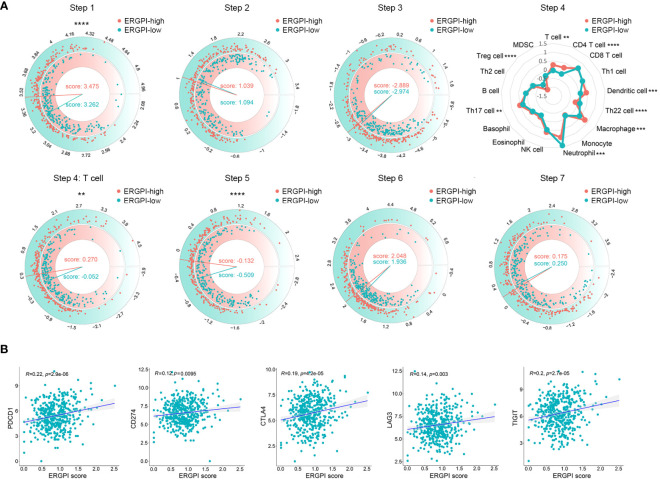
The Cancer-Immunity Cycle of high and low subgroups. **(A)** The release of cancer cell antigens (Step 1). Cancer antigen presentation (Step 2). Priming and activation (Step 3). Trafficking of immune cells to tumors (Step 4). Trafficking of T cells to tumors (Step 4). Infiltration of immune cells into tumors (Step 5). Recognition of cancer cells by T cells (Step 6). Killing of cancer cells (Step 7). **(B)** Correlation analysis between ERGPI and immune checkpoints (*CD274*, *CTLA4*, *LAG3*, *PDCD1* and *TIGIT*) in COAD patients. **p < 0.01, ***p < 0.001, ****p < 0.0001.

The anti-tumor immune response is a gradual process known as the Cancer-Immunity Cycle (29). We obtained the characteristics of the Cancer-Immunity Cycle from the TIP database and used them to analyze different ERGPI subgroups (**Figure 5A**). We found that the ERGPI-high subgroup had a greater ability to release cancer antigens compared to the ERGPI-low subgroup. For trafficking immune cells to tumors, the trafficking abilities of T cell, CD4 T cell, dendritic cell, macrophage and Th17 cell were more capable in the ERGPI-high subgroup, while Th22 cell, neutrophil and Treg cell were more capable in the ERGPI-low subgroup. In addition, infiltration of immune cells into tumors was also higher in the ERGPI-high subgroup. However, there was no differences between the different ERGPI subgroups in cancer antigen presentation, priming and activation, recognition of cancer cells by T cells and killing of cancer cells. We further analyzed the correlation between ERGPI and immune checkpoint genes, including PD-L1 (CD274), CTLA-4, lymphocyte activation gene 3 (LAG-3), PD-1 (PDCD1), T-cell immunoglobulin and immune receptor tyrosine-based inhibitory motif domain (TIGIT). There were significant positive correlations between ERGPI and these immune checkpoint genes (**Figure 5B**).

### Knockdown of *LEP*, *NGF* or *PCOLCE2* inhibited cell proliferation and EMT

**Figure 6:**
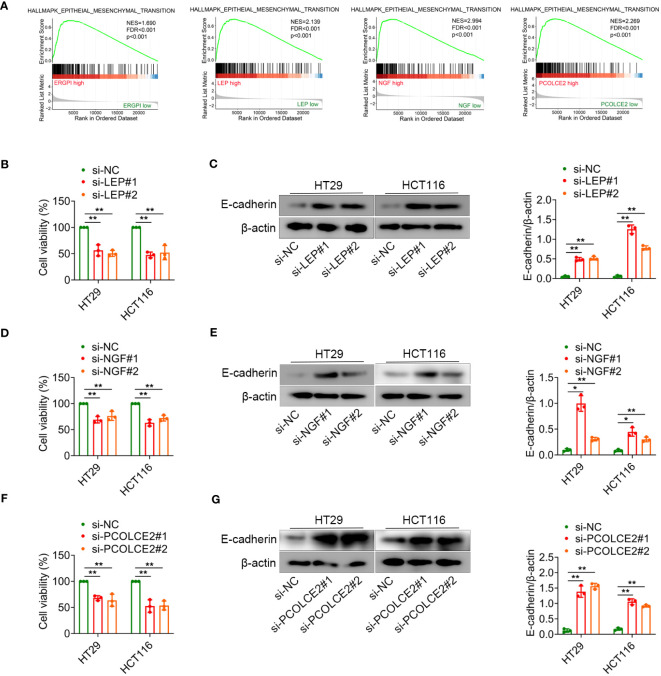
*LEP*, *NGF* or *PCOLCE2* deficiency reduces cell proliferation and EMT. **(A)** GSEA analysis for the EMT-related signature. **(B–G)** HT29 and HCT116 cells were transfected with control siRNR (si-NC), LEP siRNA (si-LEP), NGF siRNA (si-NGF) or PCOLCE2 siRNA (si-PCOLCE2). The MTT assays showed that the proliferation ability of cells. The protein expression levels of E-cadherin in cell lines (HT29 and HCT116) were determined using western blot analysis. The densities of protein were quantified using densitometry. E-cadherin were normalized to β-actin. *p < 0.05, **p < 0.01.

The EMT is the process by which epithelial cells acquire mesenchymal stem cell phenotypes under specific physiological and pathological conditions, mediating the progression and metastasis of COAD (32, 33). To better understand the effect of ERGPI and potential biomarkers on EMT, we analyzed the EMT signaling pathway using GSEA method. As shown in **Figure 6A**, high expression of ERGPI promoted EMT in COAD patients. Similar results were obtained in the *LEP*, *NGF* and *PCOLCE2* high expression groups. To further confirm the effects of *LEP*, *NGF* and *PCOLCE2* on COAD cell proliferation and EMT, siRNA was used to knockdown *LEP*, *NGF* and *PCOLCE2* in COAD cells, respectively (**Supplementary Figure S4**). We found that the knockdown of *LEP* significantly inhibited cell proliferation and up-regulated the expression level of E-cadherin protein (**Figures 6B, C**). Similar results were observed with *NGF* and *PCOLCE2* knockdown (**Figures 6D–G**). These data indicated that knockdown of *LEP*, *NGF* and *PCOLCE2* inhibited COAD cell proliferation and EMT.

### Dictamnine inhibited COAD progression in xenografts

**Figure 7:**
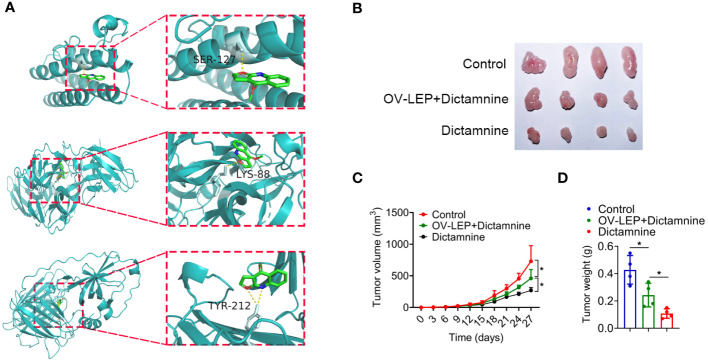
Dictamnine binds to these three proteins based on molecular docking. **(A)** Molecular docking analysis of dictamnine with LEP, NGF and PCOLCE2. **(B, C)** *Ex vivo* images of resected **(B)** and growth curves of tumor volume **(C)**. **(D)** Tumor weight. *p < 0.05.

Dictamnine is a drug component with anti-tumor potential. To further explore whether the combination of dictamnine with LEP, NGF and PCOLCE2 could exert anti-tumor effects, we performed molecular docking of dictamnine with LEP, NGF and PCOLCE2, and the predicted results indicated that dictamnine could access the potential binding pocket of LEP, NGF and PCOLCE2 (**Figure 7A**). Dictamnine forms a hydrogen bond with SER-127 of LEP with a binding energy of -5.3 kJ/mol, a hydrogen bond with LYS-88 of NGF with a binding energy of -6.2 kJ/mol, and a hydrogen bond with TYR-212 of PCOLCE2 with a binding energy of -5.7 kJ/mol. To investigate the role of dictamnine in the progression of COAD *in vivo*, we selected a potential target (LEP) for further study. Dictamnine had significant anti-tumor effects, inhibiting tumor growth and reducing tumor weight (**Figures 7B–D**). The overexpression of *LEP* inhibited the anti-tumor effect of dictamnine (**Figures 7B–D**).

## Discussion

In this study, we identified three potential biomarkers associated with ECM by combining bioinformatics analysis with experimental validation. We screened three ECM-related biomarkers (*LEP*, *NGF* and *PCOLCE2*) of COAD patients by Lasso-Cox analysis, and constructed ERGPI using the three genes. The high expression of LEP, NGF and PCOLCE2 in tumor tissues was verified by COAD clinical samples. Gene mutation, immune response and EMT signaling pathway were analyzed based on ERGPI subgroups. Knockdown of the three genes inhibited cell proliferation and EMT. We found that dicamnine binds to these three proteins based on molecular docking, which was verified by transplantation tumor models.

ECM is involved in the pathogenesis of tumors by regulating immune response, cell proliferation and metastasis (34, 35). Three ECM-related hub genes (*LEP*, *NGF* and *PCOLCE2*) associated with prognosis of COAD patients were screened by Lasso-Cox analysis. LEP plays an important role in regulating cellular metabolism. LEP stimulates tumor cell proliferation, inhibits cell apoptosis and promotes angiogenesis by binding to its receptor LEPR (19, 36). Epidemiological studies support that LEP is associated with poor prognosis of COAD patients (19). NGF is an important neuropeptide in the family of neurotrophic factors. The high expression of NGF is closely related to the proliferation, invasion and migration of tumor cells. It has been shown that NGF promotes the proliferation and metastasis of COAD cells by regulating the expression of microRNA (8). Tumor target drug gastrin-releasing peptide receptor (GRPR) antagonist RC-3095 reduces the secretion of NGF in COAD cells, suggesting that the reduction of neurotrophin secretion is a potential mechanism for the anti-proliferation effect of GRPR antagonists (37). PCOLCE2, as a collagen-binding protein, is considered to be the mediator of protein matrix. PCOLCE2 has been identified as a novel biomarker for the diagnosis and prognosis of COAD patients (38). In addition, Shi et al. found that PCOLCE2 could be a potential prognostic biomarker for identifying metastasis in COAD patients (25). We reported that LEP, NGF and PCOLCE2 were highly expressed and associated with poor prognosis in COAD patients. These biomarkers need to be further confirmed by larger clinical cohorts and basic studies.

To further understand the molecular characteristics of ERGPI subgroups, we explored the gene mutation profiles of different ERGPI subgroups. There were significant mutation differences between different ERGPI subgroups, the most obvious difference being that ERGPI-high patients (45%) had a higher frequency of KRAS mutations than ERGPI-low patients (38%). It is well known that KRAS gene inhibits the growth of tumor cells. However, once KRAS mutation occurs, it will continuously stimulate cell growth and disturb the growth rule, which will lead to the occurrence of tumors (39, 40). KRAS mutation is associated with the metastasis of COAD patients, and the 5-year survival rate of metastatic COAD patients is only 12 to 14% (40–42). Therefore, the poor prognosis of ERGPI-high patients may be related to the higher frequency of KRAS mutation.

Immune cells and immune checkpoints play an important role in the occurrence and development of tumors (43–45). To further analyze the immunological characteristics of ERGPI subgroups, we investigated the relationship between ERGPI and immune response. Our analysis results showed that the ERGPI-high subgroup had a stronger ability to release antigens to cancer cells, which may be caused by more gene mutations. Interestingly, the trafficking of immune cells to tumors and infiltration of immune cells into tumors in the ERGPI-high subgroup were significantly better than those in the ERGPI-low subgroup. Previous studies have shown that high expression of immune checkpoints (PD-L1, CTLA-4, LAG-3, PD-1, TIGIT) as potential immunotherapy targets is associated with better immunotherapy efficacy (46–48). We found positive correlations between ERGPI and these immune checkpoints. Therefore, these results suggest that COAD patients with high ERGPI expression are more likely to benefit from immunotherapy.

Our understanding of the role of potential biomarkers (*LEP*, *NGF* and *PCOLCE2*) in COAD is still limited, and further research is essential to investigate the effects on other COAD cell lines and tumors derived from COAD patients to enhance the validity of the findings. In addition, the regulation mechanism of potential biomarkers on tumor immune response and EMT needs to be further studied to collect more convincing data to support our findings.

## Conclusion

In conclusion, we identified three potential biomarkers (*LEP*, *NGF* and *PCOLCE2*) associated with ECM based on bioinformatics analysis and experimental validation. Further study of these biomarkers can provide new ideas and basis for understanding the disease progression and targeted therapy of COAD patients.

## Data availability statement

The datasets presented in this study can be found in online repositories. The names of the repository/repositories and accession number(s) can be found in the article/**Supplementary Material**.

## Ethics statement

Approval was granted by the Ethics Committee of Mudanjiang Medical University. The studies were conducted in accordance with the local legislation and institutional requirements. The human samples used in this study were acquired from primarily isolated as part of your previous study for which ethical approval was obtained. Written informed consent for participation was not required from the participants or the participants’ legal guardians/next of kin in accordance with the national legislation and institutional requirements. The animal study was approved by Ethics Committee of Mudanjiang Medical University (20221013–1). The study was conducted in accordance with the local legislation and institutional requirements.

## Author contributions

YY: Data curation, Formal analysis, Writing – original draft. XY: Data curation, Formal analysis, Writing – original draft. ZC: Data curation, Formal analysis, Writing – original draft. HW: Data curation, Formal analysis, Writing – original draft. JL: Writing – review & editing, Data curation, Formal analysis. DW: Writing – original draft, Data curation, Formal analysis. PW: Writing – original draft, Data curation, Formal analysis. BL: Writing – review & editing. JM: Writing – review & editing. QY: Conceptualization, Funding acquisition, Writing – original draft, Writing – review & editing.
