# Supplementary material for: Identification of extracellular matrix-related biomarkers in colon adenocarcinoma by bioinformatics and experimental validation
Source: Front Immunol. 2024 Apr 17;15:1371584. doi: 10.3389/fimmu.2024.1371584 (PMC11061380; doi:10.3389/fimmu.2024.1371584)
Supplement: Supplementary file 1 [file DataSheet_1.doc]

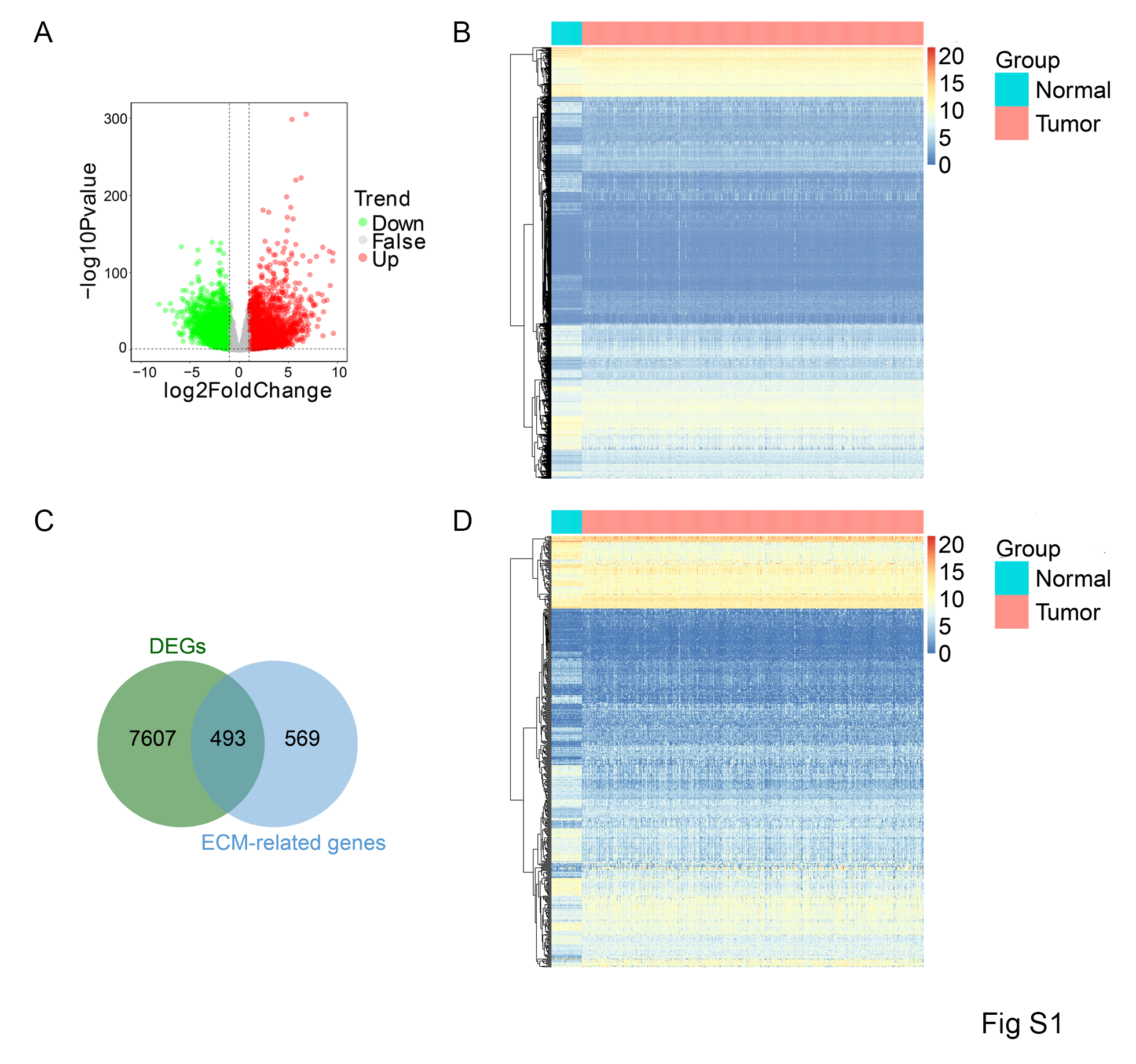


**Figure S1**

**Analysis of** **ECM-related DEGs.**

The volcano plot (A) and heatmap (B) of DEGs between normal and tumor samples in TCGA-COAD data. (C) Venn diagram of intersection genes between DEGs and ECM-related genes. (D) The heatmap of ECM-related DEGs between normal and tumor samples in TCGA-COAD data.


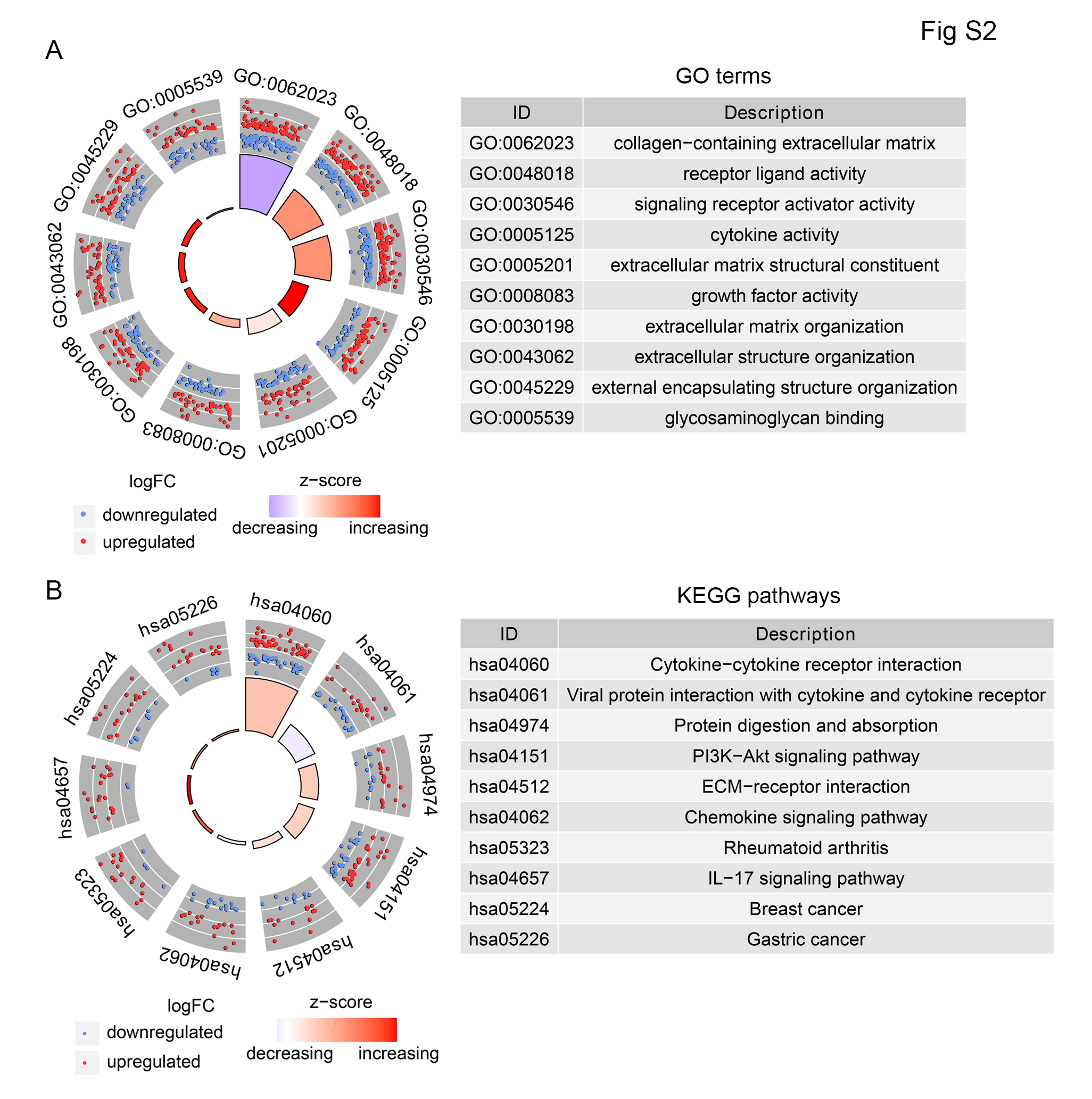


**Figure S2**

**Functional enrichment analysis.**

GO enrichment analysis (A) and KEGG enrichment analysis (B) of ECM-related DEGs.


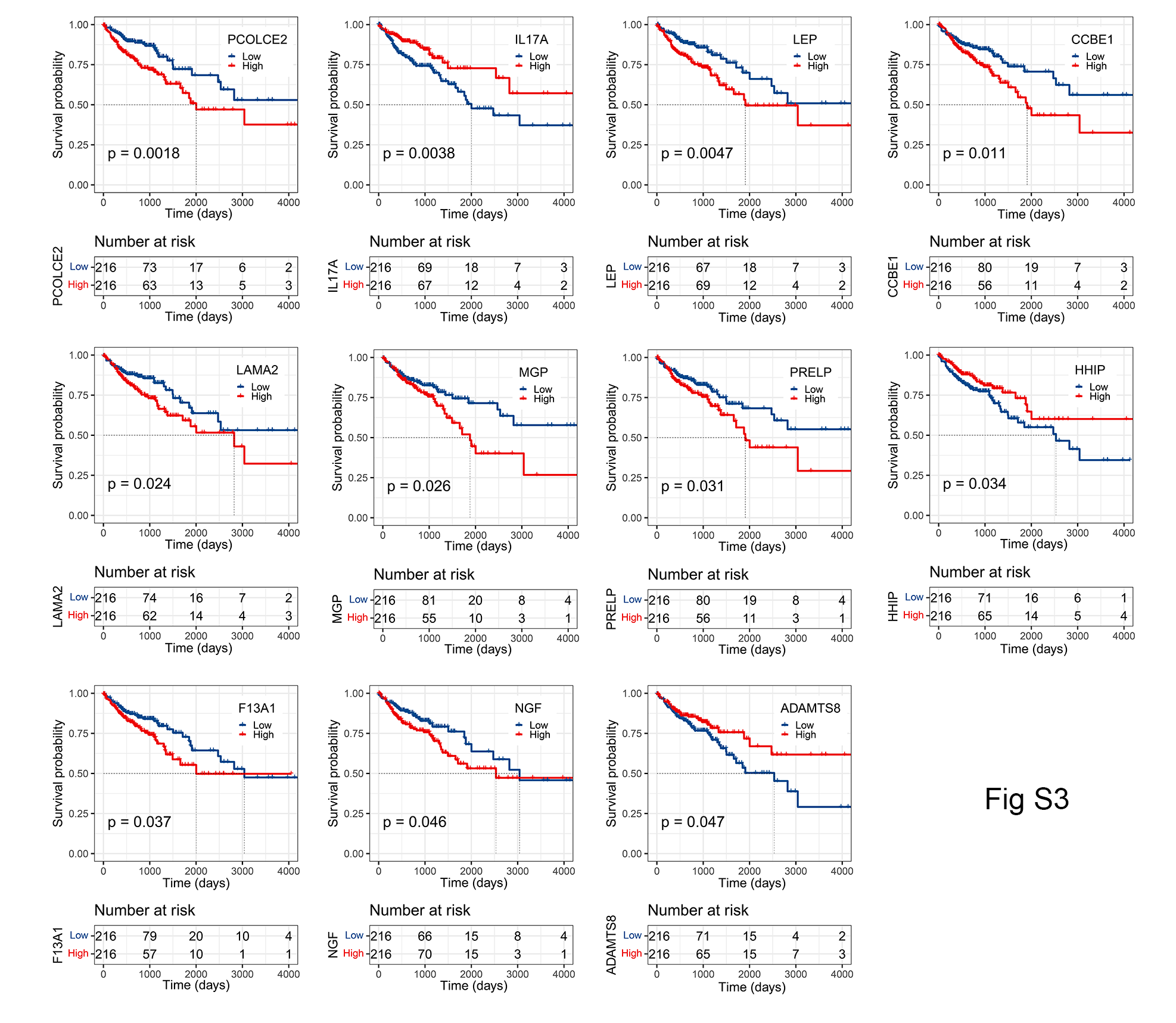


**Figure S3**

**Survival analysis of** **11 ECM-related hub genes.**

Kaplan-Meier survival analysis of 11 ECM-related hub genes in TCGA-COAD data.


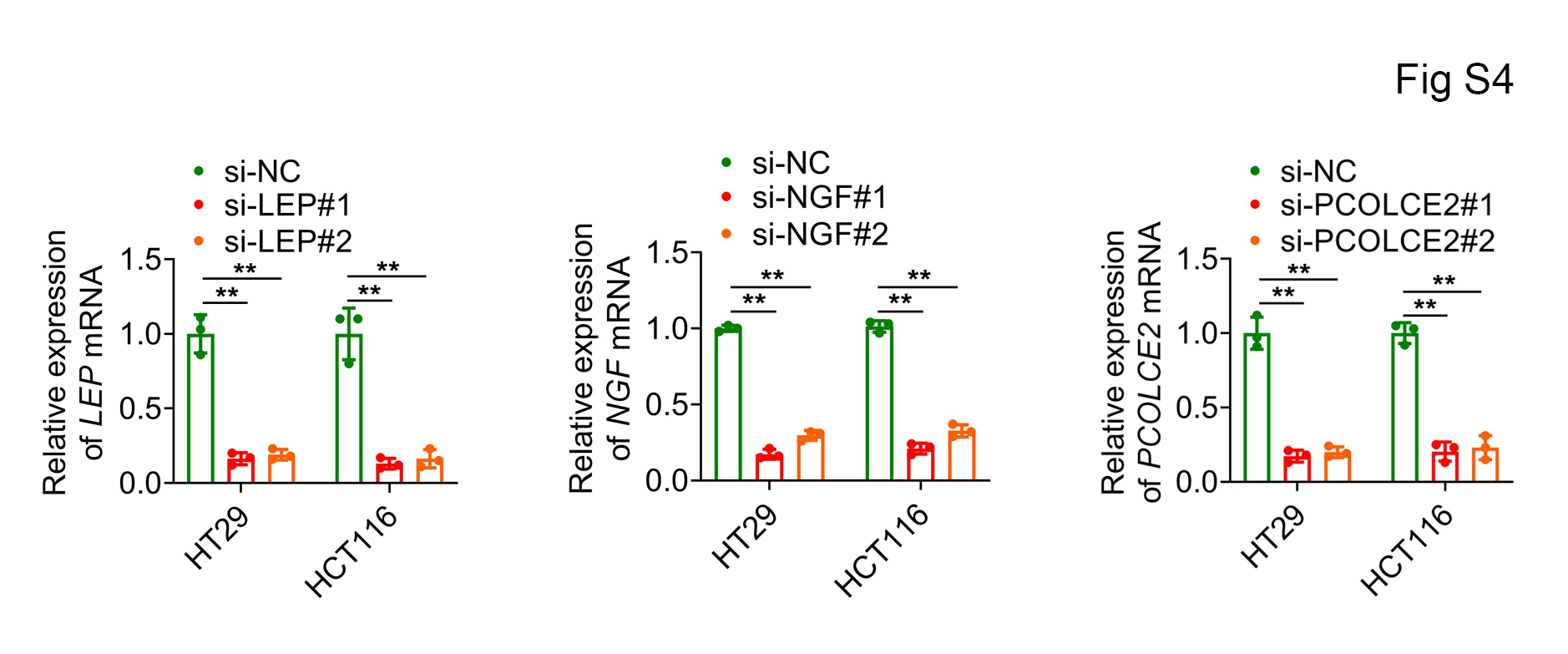


**Figure S4**

**Gene knockdown efficiency.**

The mRNA levels of *LEP*, *NGF* and *PCOLCE2* were measured using qPCR analysis. The results were normalized to *ACTB*. ***p* < 0.01.
